# Secular Trends and Socioeconomic Differentials in Menarcheal Age for South Korean Women

**Published:** 2018-09

**Authors:** Heeran CHUN, Eunhee SHIN

**Affiliations:** 1. Dept. of Health Administration, Jungwon University, Chungbuk, Korea; 2. Dept. of Nursing Science, Sangji University, Wonju, Korea

**Keywords:** Menarche, Trends, Socioeconomic factors

## Abstract

**Background:**

In this study, we assessed the secular trends and socioeconomic differentials in menarcheal age among women aged 25–64 year.

**Methods:**

Using the 5th Korea National Health and Nutrition Examination Survey (KNHANES, 2010–2012), bivariate analysis and one-way analysis of variance were used to test the statistical differences between age groups.

**Results:**

The mean age at menarche was 14.3 yr with a steep decrease in the younger cohort: from 15.9 yr in 1951–1955 to 13.1 yr in 1986–1990. Height as a proxy marker for early nutrition showed an inverse relationship with menarcheal age. The earlier menarcheal age patterns in women of higher socioeconomic position were observed according to one’s education, monthly family income, occupation, region (urban vs. rural), and parental education.

**Conclusion:**

This result suggests a fast and ongoing trend in age at menarche and the socioeconomic discrepancy among Korean women in the last four decades.

## Introduction

Menarcheal age is the starting point of sexual maturation and has significant implications for health status, growth, and reproductive life of females (1, 2). Besides, menarche is regulated by various environmental factors (3). There has been a systematic decrease in the median age at menarche in the past 160 yr among different developed countries (4–8) and the downtrend has slowed or even stopped in some European countries (9), but it is continuing in the US and Asia (10).

Several studies have suggested a relationship between socioeconomic status and decline in the median age at menarche (11, 12). Increased body size (13–15) and psychosocial stress (16, 17), found to be associated with lower socioeconomic status, could influence the alteration in age at menarche. Additionally, the relationship between maternal menarcheal age, BMI, maternal age at birth, nutrition, height and mean age at menarche has been examined (18,19).

Rapid economic and social development and quick change in the age of menarche during the last century in Korea will provide a significant case for the trend in menarcheal age. Although it was not possible to assess nutrient intakes directly, we examined the socioeconomic differences and trends in the menarcheal age based on the assumption that the socioeconomic indicators such as parental education level and household income reflect the environmental conditions.

The subjects in this study were women born between 1951 and 1990. The Korean War took place between 1950 and 1953 and Korean women born in the 1950s and 1960 have suffered from hunger and malnutrition. In contrast, women born in the 1980s spent their childhood in the period of economic development, and the fertility rate dropped below the replacement level of fertility (TFR 2.1 in 1984) at that time.

Little is known about the trend and the associated socioeconomic factors over time based on the national data. This study aimed to assess the secular trends and socioeconomic differentials in menarcheal age among women aged 25–64 yr using the 5th Korea National Health and Nutrition Examination Survey (KNHANES, 2010–2012).

## Methods

### Data and study population

The analysis of this study was based on the “the 5^th^ Korean National Health and Nutrition Examination Survey (KNHANES, 2010–2012)”. The KNHANES is a nationally representative cross-sectional survey that used a stratified, multistage probability sampling design to select household units. The survey was first initiated in 1998 to assess the health/nutrition status as well as health behavior, quality of life, and chronic diseases among Koreans. Specific information about the KNHANES was described in the published journal (20) and it was presented on the official webpage (http://knhanes.cdc.go.kr).

There were 7765 women aged 25–64 yr in the sample. After deleting all missing variables of menarcheal age, height and socioeconomic factors, we included 6830 women in the analysis to evaluate the secular trend in menarcheal age and its socioeconomic differentials. Participants aged between 25 and 64 yr were converted to the pseudo-birth-cohort from 1951 to 1990, based on the survey conducted in 2010, 2011, and 2012, respectively. Considering the ongoing physical developmental process until the early 20s, we restricted the age to more than 25 year.

### Dependent and independent variables

The dependent variable “age at menarche” was assessed based on self-reported questionnaires. The independent socioeconomic variables included educational levels(less than middle school, high school, and more than college), monthly family income (low, middle, and high), occupational classes (profession/office work, service/sales, manual, and others), place of residence (urban vs. rural), and parental education (no formal education, primary school, middle school, high school, college+, and do not know/missing). The monthly household income was categorized into tertiles after household income was divided by the square root of the household size. This was done to adjust for the differences in disposable income by the number of people in the household.

### Statistical analysis

To evaluate the secular trend in age at menarche and height, data of women aged 25 to 64 yr were stratified by their year of birth and were presented in 5-year intervals. Bivariate analysis and oneway analysis of variance (one-way ANOVA) were performed. Bonferroni’s multiple comparison tests were used to assess the statistical differences between age groups.

## Results

Table 1 presents the mean age at menarche and the mean height according to the birth cohort among South Korean women born between 1951 and 1990 (aged 25–64 yr).

**Table 1:** Menarcheal age and height according to age group, The 5th KNHANES (2010–2012)

Birth cohort^*^	***Age (years)***	***Menarcheal age***	***SD***	P^***^	***Height***	***SD***	P^***^
1951–1955	60–64	15.9	1..9	A	154.9	5.4	F
1956–1960	55–59	15.4	2.0	B	156.3	5.2	E
1961–1965	50–54	14.9	1.7	C	157.5	5.3	D
1966–1970	45–49	14.1	1.5	D	158.5	5.2	C
1971–1975	40–44	13.8	1.5	E	159.7	5.2	B
1976–1980	35–39	13.5	1.5	F	160.6	5.3	A
1981–1985	30–34	13.2	1.7	GF	161.5	5.5	A
1986–1990	25–29	13.1	1.7	G	161.3	5.7	A
Total	All	***14.3***	2.0		***158.5***	***5.7***	
Median		***14.0***			***158.5***		
Weighted mean, SE		***14.3, 0.03***			***158.7, 0.09***		

* Birth year was calculated according to the respondent’s age at the time of survey year 2010–2012 //

***: Means with different alphabet letters are significantly different by Bonferroni’s multiple comparison tests (menarche: F=395.01 *P*<.0001; height: F=199.55 *P*<..0001)

The mean age at menarche was 14.3 yr with a steep decrease in the younger cohort: by 2.8 yr from 15.9 yr in 1951–1955 to 13.1 yr in 1986–1990. Height as a proxy marker for early nutrition showed an inverse relationship with menarcheal age across birth cohorts. Height increased by 6.4 cm from 154.9 cm in 1951–1955 to 161.3 cm in 1986–1990. Table 2 shows the earlier menarcheal age according to a higher socioeconomic position. A positive association was observed according to one’s education, monthly family income, occupation, region, and parental education.

**Table 2:** Menarcheal age according to own and parental socio-economic conditions and birth cohort, The 5th KNHANES (2010–2012)

***Variable***	***n***	***%***	***1951 – 1990 Aged 25–64 (n= 6830)***		***Younger 1971 – 1990 (n=3134)***		***Older 1951 – 1970 (n= 3696)***	
			***Mean***	***P***	***Mean***	***P***	***Mean***	***P***
**Total**	6830	100	14.3		13.5		15.1	
**Education**								
<Middle school	1524	22.3	15.2	A^***^	14.3	A^***^	15.7	A^***^
High school	2708	39.7	14.3	B	13.7	B	14.8	B
>College	2598	38.0	13.8	C	13.3	C	14.3	C
**Household income**								
Lowest- Q1	2329	34.1	14.5	A^***^	13.6	A^***^	15.3	A^***^
Q2	2194	32.1	14.3	B	13.5	B	15.1	A
Highest- Q3	2307	33.8	14.1	C	13.3	B	14.8	B
**Occupational class**								
Professional/office work	1573	23.0	14.2	A^***^	13.4	A^**^	14.7	A^***^
Service/sales	1112	16.3	14.6	B	13.6	AB	15.3	B
Manual	1067	15.6	14.7	B	13.8	B	15.3	B
Not working	3078	45.1	14.2	A	13.4	A	14.9	A
**Region**								
Urban	5753	84.2	14.3^***^		13.5	ns	15.0^***^	
Rural	1077	15.8	14.6		13.6		15.3	
		%	1951 – 1990		1971 – 1990		1951 – 1970	
Father’s education^***^								
No formal education	745	10.9	14.7	A^***^	13.6	ABC^***^	15.3	A^***^
Primary school	1928	28.2	14.4	B	13.4	BC	15.1	A
Middle school	1172	17.2	14.4	B	13.6	AB	15.1	A
High school	1567	22.9	14.2	C	13.5	BC	14.7	B
>College	859	12.6	13.9	D	13.2	C	14.4	B
Don’t know	559	*8.2*	*14.7*	A	*13.9*	A	15.3	A
Mother’s education ***								
No formal education	1190	17.4	14.7	A^***^	13.7	ABC^***^	15.3	A^***^
Primary school	2595	38	14.3	B	13.5	BC	15.0	B
Middle school	1142	16.7	14.2	B	13.5	AB	14.9	B
High school	1179	17.3	14.2	BC	13.4	BC	14.7	BC
>College	303	4.4	13.9	C	13.2	C	14.0	C
Don’t know	421	*6.2*	*14.8*	A	*13.9*	A	15.4	A

- * *P*<.05,

** <.01,

*** <.0001,

NS: Not Significant //- Group differences in age-adjusted menarche by ANCOVA analysis - Means with different alphabet letters are significantly different by Bonferroni’s multiple comparison tests

One’s own education most displayed a socioeconomic group difference in menarcheal age. The age-adjusted menarcheal age was 15.2 yr among women with less than middle school education, compared to the age-adjusted menarcheal age of 13.8 yr among female college graduates, and there was a gap of 1.4 yr. Group differences in menarcheal age according to the socioeconomic indicators were significant; 0.4 yr according to household income (high vs low by the tertile group), 0.5 yr according to occupational class (Professional vs. manual work), 0.3 yr according to the region (urban vs. rural), and 0.8 yr according to both the father’s and mother’s education (college + vs. no formal education). Age-stratified analysis still showed statistical significance, except for the regional difference among women in the younger cohort (1971–1990). The socioeconomic gap in menarcheal age was wider among the older cohort (born from 1951–1970) than among the younger cohort (born from 1971–1990), showing the most substantial gap according to the mother’s education (a 1.3-year gap between college or more and no formal education).

## Discussion

We examined the secular trend in age at menarche using a representative sample of Korean women and the socioeconomic differences. The mean age at menarche decreased from 15.9 yr between 1951 and 1955 to 13.1 yr between 1986 and 1990. Our result supported the claim of consistent socioeconomic discrepancy among Korean women in the last four decades; the earlier menarcheal age patterns in women of higher socioeconomic position were observed according to one’s education, monthly family income, occupation, region (urban vs. rural), and parental education.

Concerning for the mean age at menarche and the downward trend, our results showed both similar and different patterns from those in the previous studies. The earliest mean age at menarche has been found to range from approximately 16.8 yr in 1920 to 12.7 yr in 1986 (19) and from 16.9 yr between 1920 and 1925 to 13.8 yr between 1980 and 1985 (18). In our study, the age at menarche in girls born between 1981 and 1985 was 13.2 yr and it was slightly lower than the age reported in a previous study (18), but it was substantially higher than the age reported in another study (19). This discrepancy could be attributed to the source of different populations. The Korean National Health and Nutrition Survey data were analyzed as we did in our study, but Hwang stated that they selected subjects from the Ansan Health Study sample. Ansan is an industrialized and urbanized region in Korea. Accordingly, this result might stem from the fact that a larger number of girls from the rural or urban area in this birth year group were evaluated in our study.

Several studies have shown a systematic decline in mean age at menarche, indicating values of 0.21 yr per decade in Brazilian women between 1920 and 1970 (21), 0.24 yr per decade in Norwegian women between 1830 and 1960 (22), 0.12 yr per decade in Dutch women between 1955 and 1997 (6) 0.50 yr per decade in black South African girls between 1956 and 2004 (23), 0.7 yr per decade in Chinese women over a 40-year period (2). In this study, the downward trend was 0.7 yr per decade, consistent with previous studies in Korean women, showing a downward trend of 0.68 yr per decade (18). Further studies are needed to clarify this secular trend in Korean women already reached their earliest age of menarche or to assess whether the decrease in age at menarche is continuing.

We verified that height as a proxy marker for early nutrition showed an inverse relationship with the menarcheal age across birth cohorts. Age at menarche is dependent on height (25) since nutrition is an essential determinant of age at menarche. A possible explanation for this relationship is that higher dietary energy intake is associated with higher height and an earlier age at menarche. Approximately 95% of height had been achieved at the age at menarche compared to the mature height (26). Moreover, menarcheal girls consumed more dietary energy than premenarcheal girls after adjustment for age (18).

“The socio-economic status-dependent variability of growth and maturation rate has been observed in various populations, both in highly developed and developing countries” (27, 28). Socioeconomic variables such as parental education, region (urban or rural), one’s education, and so on are strongly correlated with dietary habits, lifestyle, and healthcare (29, 30). In the present study, a positive association was observed according to one’s education, monthly family income, occupation, region, and parental education. One’s education and father’s education are considered to be the indicators of the economic status, while mother’s education is associated with knowledge of diet and hygiene. “Higher socioeconomic status improves nutrition and consequently favors early menarche” (2, 9). In this regard, one explanation is that a critical proportion of body fat (17%–22%) may be required to trigger menarche (14). However, in the last few decades, the influence of socio-economic status distinguished by profession or education of parents on the age at menarche seems to have diminished in developed countries. Because when living conditions are good, further improvement or slight difference does not affect the biological development (31, 32). Similarly, the difference in age at menarche between social strata has become non-significant in developing countries due to the uniformity of living conditions (32). South Korean women born in the 1950s had experienced the Korean War (1950–1953) and they mostly lived in poverty. However, the gross domestic product of Korea increased by approximately 900% from 1955 to 1986 after the Seoul Asian Games in 1986 and the Seoul Olympics in 1988. Therefore, the schoolgirls born in the 1980s had rapidly improved nutrition and living standards. Although the findings of this study are in agreement with the literature, wherein high socio-economic status is shown to be associated with earlier menarche, further studies are necessary for South Korean women born after the 1980s since living conditions have improved.

The menarcheal age of urban girls was lesser than that of rural girls in a previous study (33). Likewise, in this study, women living in the urban area had earlier menarche. A possible explanation for this occurrence is that the percentage of people with higher socio-economic status is greater in the urban area than in the rural area (34).

A limitation of this study is that we used a cross-sectional design which is vulnerable to recall bias. However, the recall method of age at menarche is reliable and valid (34, 35). Another limitation of this study is that the cross-sectional design prohibits conclusions about a causal relationship between age at menarche and socioeconomic status.

## Conclusion

This study shows the secular trend in age at menarche and its association with socioeconomic status among South Korean women. Further studies are necessary to assess the trend in menarcheal age and its relationship with South Korea’s socioeconomic development, considering the industrial growth after the 1990s.

## Ethical considerations

Ethical issues (Including plagiarism, informed consent, misconduct, data fabrication and/or falsification, double publication and/or submission, redundancy, etc.) have been completely observed by the authors.
